# Is there a standardized practice for the development of international ulcerative colitis and Crohn’s disease treatment guidelines?

**DOI:** 10.1093/gastro/goab009

**Published:** 2021-03-29

**Authors:** Alexander Goldowsky, Rohan Sen, Gila Hoffman, Joseph D Feuerstein

**Affiliations:** 1 Department of Medicine, Beth Israel Deaconess Medical Center, Boston, MA, USA; 2 Department of Gastroenterology and Hepatology, Beth Israel Deaconess Medical Center, Boston, MA, USA

**Keywords:** Crohn’s disease, ulcerative colitis, inflammatory bowel disease, guidelines, conflicts of interest

## Abstract

**Background:**

Guidelines are published by international gastroenterology societies regarding the management of ulcerative colitis (UC) and Crohn’s disease (CD) to help clinicians to provide high-quality patient care. We examined the guidelines for the quality and strength of evidence used to develop the recommendations, methods for grading evidence, differences in disease-specific recommendations, conflicts of interest, and plans for guideline updates.

**Methods:**

A systematic search was performed on PubMed using “ulcerative colitis,” “Crohn’s disease,” and “guidelines” in April 2019. International gastroenterology society websites were searched for UC- and CD-specific guidelines. Guidelines from 12 societies were examined by two authors. Chi-squared tests were used for comparing evidence-level grades, strength of recommendations, and reported conflicts of interest. Linear-regression modeling was used to evaluate the relationship between the number of authors and the number of recommendations in a given guideline.

**Results:**

Of 28 guidelines reviewed, 25 (89%) used a total of three different systems to grade the level of evidence and 2 (7%) used an unknown system. Three (11%) reviewed guidelines did not provide a conflict-of-interest statement, while three (11%) provided a timeline for guideline updates. Of 1,265 total statements examined, 246 (19%) reported no grade of evidence quality or explicitly stated that the recommendation was based on “expert opinion.” One hundred and thirty-five (22%) UC recommendations were noted to be “weak/conditional” and 95 (16%) did not have a recommendation strength. Two hundred and forty-two (37%) CD recommendations were noted to be “weak/conditional” and 151 (23%) did not have a recommendation strength.

**Conclusion:**

The majority of UC and CD guidelines are based on a low/very low quality of evidence and are further weakened due to the lack of homogeneity in specific aspects of management recommendations as well as conflicts of interest.

## Introduction

Clinical practice guidelines (CPGs) were first formally defined by the Institute of Medicine (IOM) in 1990 [1]. The definition was updated in 2011 and states that they are “statements that include recommendations, intended to optimize patient care, that are informed by a systematic review of evidence and an assessment of the benefits and harms of alternative care options” [2]. The systematic-review portion of this process was targeted by the Appraisal of Guidelines for Research and Evaluation II (AGREE) in 2009 (updated in 2017), such that there could be a more formalized framework to assess the quality of guideline evidence, provide a standardized strategy for guideline development, and inform how guidelines are reported [3]. Prior to the IOM, individual societies and national organizations employed varying processes in guideline development.

In the 2011 IOM update, the organization further described standards for developing trustworthy CPGs. These include the following: funding transparency, managing/disclosure of conflicts of interest, having a multidisciplinary development group that includes patient representatives, using a systematic-review process, establishing evidence foundations and rating the strength of recommendations, standardizing the articulation of the recommendation, performing an external review, and having a update schedule for recommendations [2].

The first review of inflammatory bowel disease (IBD) international guidelines was performed in 2013 [4]. It found that nearly half of IBD recommendations were based on expert opinion or no evidence. In addition, a majority of international guidelines at the time failed to disclose conflicts of interest (COI). These guidelines also did not delineate a time frame for which regular updates would occur. Finally, there was substantial disagreement between guidelines regarding the best practices for managing various aspects of IBD patient care [4]. These were not isolated problems in the IBD literature, as studies in other fields (cardiology, infectious disease) yielded similar results [5–7].

Since 2013, unfortunately not a great deal has changed. A 2019 study of American Heart Association/American College of Cardiology guidelines again found significant variation in levels of evidence supporting various interventions and that there was very little high-quality evidence [8]. Similar findings were seen in rheumatology, endocrinology, and hepatology [9–11].

There has not been a review of more recent IBD guidelines to assess whether there has been a change in compliance with IOM CPG standards. We performed a systematic review of major international gastroenterology and IBD society guidelines specifically on the topic of Crohn’s disease (CD) and ulcerative colitis (UC) that have been published in the literature and on the websites from these societies. Our primary aim was to assess the overall quality of evidence cited in formulating the recommendations and the strength of these recommendations. As secondary aims, we also sought to determine the methods used to grade the evidence, evaluate for differences in recommendations or grades of evidence, gauge potential COI, delineate plans for guideline updates, and highlight opportunities for improvement.

## Methods

### Guidelines

A systematic search was performed on PubMed including Mesh terms for UC and guidelines, as well as CD and guidelines, in April 2019. In addition, major international gastroenterological society websites were also examined for the presence of UC- and CD-specific guidelines. Guidelines that were not present in the systematic PubMed search nor posted on the societies’ guidelines webpage were excluded in this analysis. Pediatric guidelines were excluded in an effort to distinguish pediatric IBD guidelines from adult IBD guidelines. Societies with no available English guidelines were excluded in order to prevent errors in translation. Guidelines were also excluded if they were not specific for CD or UC (i.e. categorized as general “IBD guidelines”), as these disease states are unique and CPGs differ with regard to disease management. Finally, in the event of duplicate guidelines with the same title, the earlier guideline was excluded from the analysis.

The following societies were ultimately included in the final analysis: American College of Gastroenterology (ACG), American Gastroenterological Association (AGA), Asia Pacific Association of Gastroenterology, National Institute for Health and Care Excellence (NICE), Canadian Association of Gastroenterology (CAG), Crohn’s and Colitis Foundation of America (CCFA), European Crohn’s and Colitis Organisation (ECCO), Gastroenterological Society of Australia, Indian Society of Gastroenterology, Japanese Society of Gastroenterology, Korean Association for the Study of Intestinal Diseases, New Zealand Society of Gastroenterology, and the Spanish Working Group (GETECCU) [12–40].

In the category of UC guidelines, we identified 97 guidelines published by 31 gastroenterology societies. Nineteen societies were excluded based on the exclusion criteria mentioned above. In the category of CD guidelines, we identified 118 guidelines published by 28 international societies. Twenty societies were excluded based on the exclusion criteria mentioned above. Finally 12 societies’ UC guidelines and 9 societies’ CD guidelines were reviewed. Of note, AGA includes both guidelines and a technical review. The technical review was analysed with regard to authorship and COI only (see below) [15, 17, 19]. The Asia Pacific Association of Gastroenterology CD guidelines consisted of two parts of which only Part 2 was analysed, as it discussed management [38].

Guidelines that met inclusion criteria were further examined to determine whether any grading system was used to assess the level of evidence for the recommendations. The quality of the level of evidence supporting the recommendations was evaluated for each individual guideline. In addition, the strength of recommendations was categorized. The guidelines and websites were also reviewed for any comment regarding planned updates to their current guidelines. The guidelines were examined individually, in aggregate by society, between societies, and in an overall analysis of all published IBD practice guidelines.

### Levels of evidence

Multiple systems were utilized to the grade level of evidence by the different societies. These included GRADE [41], Oxford Levels of Evidence [42], Canadian Task Force on the Periodic Health Examination, and the traditional ABC(D) system [5, 6]. It should be noted that, unlike the Oxford Levels of Evidence, GRADE methodology not only incorporates the types of studies being applied to a given recommendation (i.e. randomized clinical trial, cohort study, case series, expert opinion), but also asks questions about values and preferences, risks and benefits, resource implications, equity, and feasibility, thus adding additional rigor.

In an effort to standardize the reporting of level of evidence, the following categorizations were used (based on the GRADE and ABC(D) systems) similar to prior publications [43, 44]:

***High quality of evidence***: Oxford level 1, GRADE high quality, A level, I.

***Moderate quality of evidence***: Oxford level 2, GRADE moderate quality, B level, II-1, II-2, II-3.

***Low and very low quality of evidence***: Oxford levels 3, 4, and 5, GRADE low and very low quality, C and D level, III.

In addition, recommendations were further categorized as strong, weak/conditional, and no quality provided/expert opinion. If the guideline contained a clear recommendation as indicated by bullet point (or similar), or a recommendation was separated out from the remainder of the text and there was no accompanying grade or level of evidence noted, then it was assigned to this last category.

### Comparison of recommendations

In an effort to further delineate variation between societies’ guidelines, representative recommendations for both UC and CD were analysed in greater detail. For UC, recommendations regarding initial and surveillance screening colonoscopy, as well as colonoscopy technique (i.e. chromoendoscopy), were chosen as representative topics to be reviewed for consistency and discrepancy across guidelines. For CD, this was done using recommendations on the medical management of moderate/severe CD, fistulizing CD, and post-operative CD management. CD guidelines were also assessed for the presence of material on anti-integrins and anti-interleukins. If there were inconsistencies, the recommendations and level of evidence used to support the recommendations were further analysed.

### COI

All guidelines/websites were evaluated to determine whether potential COI were disclosed. If COI were present, the guideline was reviewed to determine the total number of authors with COI, as well as whether the primary author and principal investigator (last author) had COI. COI that were determined to be relevant included the following: advisory board, speaker's bureau, consulting, and industry-sponsored continuing medical-education activities. Government and non-profit awards were not considered COI and were excluded from analysis. The COI were assessed by individual society, between societies, and in aggregate.

### Review of the guidelines and data analysis

All guidelines were reviewed by two authors (A.G. and R.S.) for the use of a grading system for the quality of evidence behind recommendations, the specific system by which the evidence was graded, the clarity of the document layout, the presence of COI, and evaluating similarities and discrepancies between CD and UC recommendations.

Chi-squared tests were used for comparing evidence-level grades, strength of recommendations, and COI reported between societal guidelines for all international organizations analysed. Linear-regression modeling was used to evaluate the relationship between the number of authors and the number of recommendations in a given guideline. A *p*-value of 0.05 was considered significant. Analysis was done using SAS.

## Results

### Guidelines grading of the quality of evidence

A total of 215 guideline documents were reviewed for inclusion in this study. After exclusion criteria were applied, 28 guidelines (including technical reviews from the AGA) were further analysed. Twenty-seven (96%) guidelines graded the quality of evidence behind their recommendations. The only group that did not was the New Zealand Society of Gastroenterology [36].

### Levels of evidence

After excluding AGA technical reviews, 21 of the original 28 guidelines were able to be merged into the grading system for evidence quality used in this study. The 21 guidelines had a total of 1,265 recommendations. Of these, 246 (19%) reported no grade of evidence quality or explicitly stated that the recommendation was based on “expert opinion.”

For UC, a total of 604 recommendations were analysed for evidence quality. Ninety (15%) recommendations were supported by high-quality evidence, whereas 170 (28%) were supported by moderate-quality evidence and 273 (45%) by low- or very-low-quality evidence. The breakdown by society on grading of the level of evidence by society is shown in Table 1. The proportion of high-quality evidence across societies significantly differed (*P *<* *0.001).

**Table 1.** Ulcerative colitis summary of findings

Characteristic	ACG	Asia Pacific Association of Gastroenterology	NICE	CAG (mild severe outpts)	CAG (severe hospitalized patients)	Gastroenterological Society of Australia	ECCO Part 1	ECCO Part 2	Indian Society of Gastroenterology	Korean Association for the Study of Intestinal Diseases	Mexican consensus group	National Clinical Guideline Centre (UK)	New Zealand Society of Gastroenterology	Spanish working group (GETECCU)
Year of publication	2019	2010	2013	2015	2012	2016	2017	2017	2012	2017	2018	2013	2015	2013
Number of authors	5	18	14	10	9	21	17	13	29	10	5	14	8	5
COI reports in paper or on website	Yes/Yes	No	Yes	Yes	Yes	Yes	Yes	Yes	No	Yes	Yes	Yes	Yes	Yes
First/senior author with COI	Yes/Yes	–	–	Yes/Yes	Yes/Yes	No/Yes	Yes/Yes	No/–	–	No/No	Yes/Yes	Yes/Yes	Yes/Yes	Yes/Yes
Percentage of authors with COI	80%	–	71%	90%	78%	43%	71%	54%	–	0%	40%	71%	25%	100%
Literature review performed/reported	Yes/No	Yes/yes	Yes/Yes	Yes/Yes	Yes/Yes	Yes/No	Yes/No	Yes/No	Yes/No	Yes/No	Yes/No	Yes/Yes	No/No	Yes/No
Evidence graded/method	Yes/ GRADE	Yes/based on Canadian Task Force on the Periodic Health Examination	Yes/ GRADE	Yes/ GRADE	Yes/ GRADE	Yes/ Oxford	Yes/ Oxford	Yes/ Oxford	Yes/ ABCD	Yes/ GRADE	Yes/ GRADE	Yes/ GRADE	No/-	Yes/ GRADE
Number of total recommendations	49	32	40	34	21	33	124	88	37	46	68	–	–	32
Number (%) of recommendations with high-quality evidence	2 (4%)	–	–	3 (9%)	2 (10%)	1 (3%)	8 (6%)	34 (39%)	16 (43%)	9 (20%)	10 (15%)	–	–	5 (16%)
Number (%) of recommendations with moderate-quality evidence	22 (45%)	–	–	10 (29%)	11 (52%)	3 (9%)	34 (27%)	26 (30%)	14 (38%)	13 (28%)	25 (37%)	–	–	12 (38%)
Number (%) of recommendations with low- or very-low-quality evidence	25 (51%)	–	–	21 (62%)	8 (38%)	29 (88%)	82 (66%)	29 (32%)	7 (19%)	24 (52%)	33 (49%)	–	–	15 (47%)
Number (%) of strong recommendations	34 (69%)	–	–	28 (82%)	21 (100%)	6 (18%)	–	–	16 (43%)	36 (78%)	11 (16%)	–	–	17 (53%)
Number (%) of weak/conditional recommendations	15 (31%)	–	–	6 (18%)	0 (0%)	23 (70%)	–	–	14 (38%)	10 (22%)	54 (79%)	–	–	13 (41%)
Number (%) of recommendations with no quality provided/expert opinion	0 (0%)	–	40 (100%)	0 (0%)	0 (0%)	4 (12%)	29 (23%)	10 (11%)	9 (24%)	0 (0%)	3 (4%)	–	–	0 (0%)
Funding of manuscript from industry	No	Yes	No	Yes	Yes	Yes	No	No	Yes	No	Yes	No	No	Yes
Reports of external review of manuscript	No	No	Yes	Yes	Yes	Yes	Yes	Yes	No	No	No	Yes	No	Yes
Patient representative included	No	No	Yes	No	No	No	No	No	No	No	No	Yes	No	Yes
Timeline for future review or update provided	No	No	Yes	No	No	No	No	No	No	No	No	No	No	No

ACG, American College of Gastroenterology; AGA, American Gastroenterological Association; NICE, National Institute for Health and Care Excellence; CAG, Canadian Association of Gastroenterology; ECCO, European Crohn’s and Colitis Organisation; COI, conflicts of interest.

For CD, a total of 661 recommendations were analysed for evidence quality. Ninety-three (14%) recommendations were supported by high-quality evidence, whereas 157 (24%) were supported by moderate-quality evidence and 353 (53%) by low- or very-low-quality evidence. The breakdown by society on grading of the level of evidence by society is shown in Table 2. Similarly to the UC recommendations, the proportion of high-quality evidence across societies significantly differed (*P *<* *0.001).

**Table 2.** Crohn’s disease summary of findings

Characteristic	ACG	AGA (guideline)	AGA (technical review)	AGA (guideline)2	AGA (technical review)2	Asia Pacific Association of Gastroen terology	NICE	CAG (fistulizing Crohn’s disease)	CAG (use of anti-TNFs)	CCFA	ECCO Part 1	ECCO Part 2	Japanese Society of Gastroen terology	Korean Association for the Study of Intestinal Diseases
Year of publication	2018	2018	2017	2013	2013	2016	2012	2018	2009	2015	2016	2016	2013	2017
Number of authors	6	6	7	5	5	26	19	24	6	2	23	19	6	10
COI reports in paper or on website	Yes	Yes	Yes	Yes	Yes	No	Yes	Yes	Yes	Yes	Yes	Yes	Yes	Yes
First/senior author with COI	Yes/Yes	No/Yes	Yes/Yes	No/No	No/Yes	–	–	Yes/Yes	Yes/Yes	No/No	Yes/Yes	Yes/Yes	No/Yes	No/No
Percentage of authors with COI	83%	0%	43%	0%	40%	–	42%	83%	100%	0%	70%	58%	67%	0%
Literature review performed/reported	Yes/No	Yes/Yes	Yes/Yes	Yes/Yes	Yes/Yes	Yes/Yes	Yes/Yes	Yes/No	Yes/No	Yes/No	Yes/No	Yes/No	Yes/No	Yes/Yes
Evidence graded/method	Yes/ GRADE	Yes/ GRADE	Yes/ GRADE	Yes/ GRADE	Yes/ GRADE	Yes/based on the Canadian Task force on the Periodic Health Examination	Yes/ GRADE	Yes/ GRADE	Yes/ GRADE	Yes/ ABCD	Yes/ Oxford	Yes/ Oxford	Yes/ Unknown	Yes/ GRADE
Number of total recommendations	60	6	6	10	10	28	45	7	29	10	90	100	202	58
Number (%) of recommendations with high-quality evidence	5 (8%)	0 (0%)	0 (0%)	2 (20%)	2 (20%)	1(4%)	–	0 (0%)	12 (41%)	0 (0%)	25 (28%)	13 (13%)	12 (6%)	21 (36%)
Number (%) of recommendations with moderate-quality evidence	28 (47%)	4 (67%)	4 (67%)	5 (50%)	5 (50%)	4 (14%)	–	0 (0%)	6 (21%)	10 (100%)	18 (20%)	36 (36%)	28 (14%)	9 (14%)
Number (%) of recommendations with low- or very-low-quality evidence	27 (45%)	2 (33%)	2 (33%)	3 (30%)	3 (30%)	11(39%)	–	7 (100%)	11 (38%)	0 (0%)	45 (50%)	49 (49%)	162 (80%)	31 (53%)
Number (%) of strong recommendations	37 (62%)	1 (17%)	1 (17%)	5 (50%)	5 (50%)	–	–	2 (29%)	–	–	–	–	32 (16%)	23 (40%)
Number (%) of weak/conditional recommendations	23 (38%)	5 (83%)	5 (83%)	4 (40%)	4 (40%)	–	–	5 (71%)	–	–	–	–	170 (84%)	26 (45%)
Number (%) of recommendations with no quality provided/expert opinion	0 (0%)	0 (0%)	0 (0%)	0 (0%)	0 (0%)	12(43%)	–	0 (0%)	–	–	27 (30%)	17 (17%)	95 (47%)	0 (0%)
Funding of manuscript from industry	No	No	No	No	No	Yes	No	Yes	Yes	No	No	No	No	No
Reports of external review of manuscript	Yes	Yes	Yes	Yes	Yes	No	Yes	Yes	Yes	Yes	Yes	Yes	Yes	Yes
Patient representative included	No	No	No	No	No	No	Yes	No	No	No	No	No	No	No
Timeline for future review or update provided	No	No	No	No	No	No	Yes	No	Yes	No	No	No	Yes	No

ACG, American College of Gastroenterology; AGA, American Gastroenterological Association; NICE, National Institute for Health and Care Excellence; CAG, Canadian Association of Gastroenterology; CCFA, Crohn’s and Colitis Foundation of America; ECCO, European Crohn’s and Colitis Organisation; COI, conflicts of interest.

### Methods utilized to grade the evidence for recommendations and format

As noted above, the methods used to grade evidence in these documents were variable. Seventeen (61%) guidelines used the GRADE system, five (18%) used the Oxford system, and two (7%) used the ABC(D) method of evidence-quality grading. One society (Japanese Society of Gastroenterology) used an unknown, non-standardized method of evidence grading [33]. NICE noted that a grading system was used, but did not document a grade for the level of evidence of their guideline recommendations [20, 21]. The Asia Pacific Association of Gastroenterology used the Canadian Task Force on the Periodic Health Examination to grade the evidence [38–40].

### Strength of recommendations

After excluding societies that did not note the strength of their recommendations in the entirety of their guidelines, the remaining subgroups (for UC and CD, respectively) were further analysed. There was a statistically significant difference in the strength of recommendations (strong, weak/conditional, none) for both UC and CD (*P *<* *0.001).

One hundred and sixty-nine (28%) UC recommendations were delineated “strong,” while 135 (22%) were denoted “weak/conditional” and 95 (16%) did not have a recommendation strength. For CD, 106 (16%) recommendations were delineated “strong,” while 242 (37%) were denoted “weak/conditional” and 151 (23%) did not have a recommendation strength. Breakdown by individual society for UC and CD can be seen in Tables 1 and 2.

### COI

Three societies did not disclose COI for their guideline authors (total of four guideline documents—Asia Pacific Association of Gastroenterology, AGA, and Indian Society of Gastroenterology) [14, 32, 38–40]. There was found to be a statistically significant difference in the percentage of guideline authors with COI among societies for both UC and CD (both *P *<* *0.001). The percentage of guideline authors with COI ranged from 0% to 100%, with a mean of 52% (standard deviation* *=* *32.2%). Fourteen (50%) first authors and 18 (64%) senior authors reported COI (Tables 1 and 2).

### Comparison of recommendations

Among the 604 UC recommendations, 169 (28%) were delineated “strong,” while 135 (22%) were denoted “weak/conditional” and 95 (16%) did not have a recommendation strength. Among the 661 CD recommendations, 106 (16%) were delineated “strong,” while 242 (37%) were denoted “weak/conditional” and 151 (23%) did not have a recommendation strength. See Tables 1 and 2 for the recommendations and the differing levels of evidence used to support the recommendations for UC and CD.

For UC, there was significant guideline variability on the timing of the initial colon-cancer screening for UC patients, ranging from no recommendation to first screening colonoscopy 8–10 years after diagnosis (Table 3). Four out of seven societies (57%) provided recommendations without a supporting grade of evidence (ACG, NICE, Australia, and ECCO). Three societies did not discuss initial screening (Asia Pacific, CAG, and Korea). Recommendations for the surveillance-colonoscopy frequency in UC patients were provided by 71% of societies (five out of seven: ACG, Asia Pacific, NICE, Australia, and ECCO). Two societies had no statement on surveillance colonoscopy (CAG and Korea). Recommended colonoscopy techniques (i.e. chromo-endoscopy) were not discussed in 71% of the guidelines (five out of seven: Asia Pacific, NICE, CAG, Australia, and Korea).

**Table 3.** Differences in ulcerative-colitis recommendations by society

Society	Initiation of colon-cancer screening	Colon-cancer surveillance	Colonoscopy technique
American College of Gastroenterology	In patients with UC extending beyond the rectum should start 8 years after the diagnosis (no grade)	1- to 3-year intervals based on combined risk factors for colorectal cancer and findings on prior colonoscopies (no grade)	Dye-spray chromoendoscopy with methylene blue or indigo carmine when using standard-definition colonoscopy to identify dysplasia (strong recommendation, low quality of evidence) White-light endoscopy with narrow-band imaging or dye-spray chromoendoscopy with methylene blue or indigo carmine when using high-definition colonoscopy to identify dysplasia (conditional recommendation; low quality of evidence)
Asia Pacific Association of Gastroenterology	Not discussed	Colonoscopy advised in patients with long-standing UC not involving the rectum (“II-3 Evidence obtained from comparison between time or places with or without intervention,” Class C “There is poor evidence to support the statement but recommendation made on other ground(s)”)	Not discussed
National Institute for Health and Care Excellence	Referred to separate guideline; after 10 years in those who have ulcerative colitis, but not proctitis alone (no grade)	Referred to separate guideline; every 5 years for low-risk; every 3 years for intermediate-risk; every year for high-risk (no grade)	Not discussed
Canadian Association of Gastroenterology	Not discussed	Not discussed	Not discussed
Gastroenterological Society of Australia	Patients with long-standing colitis >8 years (no grade)	1/3/5 years based on risk level (no grade)	Not discussed
European Crohn’s and Colitis Organisation	Over 8 years following the onset of symptoms to all patients to reassess disease extent and exclude dysplasia (very low quality of evidence/no grade)	Surveillance needed for all, except proctitis (moderate quality of evidence/no grade); high-risk every year (low quality of evidence/no grade); intermediate-risk every 2–3 years (very low quality of evidence/no grade); low-risk every 5 years (very low quality of evidence/no grade)	Chromoendoscopy increases dysplasia detection (moderate level of evidence/no grade); do random and targeted biopsies if using white light (low level of evidence/no grade); use high definition when available
Korean Association for the Study of Intestinal Diseases	Not discussed	Not discussed	Not discussed

For CD, there was significant recommendation variability in the medical management of moderate/severe CD (Table 4). Four out of seven societies reviewed recommend steroids and thiopurines as first-line for the induction and maintenance of remission, with variable evidence quality (AGA, NICE, ECCO, and Korea). One society recommended combination anti-TNF+thiopurine (ACG) and one had no recommendation on this topic (CAG). In fistulizing CD, five societies recommended antibiotics with or without antitumor necrosis factors (TNFs) therapy (Asia Pacific, CAG, ECCO, and Korean). Evidence quality, though, was variable. In post-operative CD, first-line recommendations included 5-aminosalicylic acid (5-ASA), thiopurines, and anti-TNFs, with only one society prioritizing a single drug class (ACG). The supporting evidence quality was heterogeneous. Three societies discussed anti-integrins and anti-interleukins in CD management (AGA, ECCO, and Korean).

**Table 4.** Differences in Crohn’s-disease recommendations by society

Society	Moderate/severe Crohn's first-line treatment	Moderate/severe Crohn's second-line treatment	Moderate/severe Crohn's combination treatment with immunomodulator + anti-TNF	Fistulizing Crohn's first-line treatment	Fistulizing Crohn's second-line treatment	Fistulizing Crohn's combination treatment with immune modulator + anti-TNF	Post-operative Crohn's first-line treatment	Post-operative Crohn's second-line treatment	Post-operative Crohn's combination treatment with immune modulator + anti-TNF	Discussion of vedolizumab	Discussion of ustekinumab
American College of Gastroenterology	Anti-TNF + TP (moderate quality of evidence/conditional)	Anti-TNF monotherapy (moderate quality of evidence/strong)	Anti-TNF + TP (moderate quality of evidence/conditional)	Infliximab (moderate quality/strong) + antibiotics (moderate quality/strong)	TP (low quality/strong), adalimumab, certolizumab (low quality/strong)	Not discussed	Anti-TNF within 4 weeks (low quality/conditional)	TP, but not effective at preventing severe recurrence (moderate quality/strong)	Recommend to decrease immunogenicity and loss of response (very low quality/conditional)	No	No
American Gastroenterological Association	Steroids for short term (moderate quality of evidence/strong); TP for steroid sparing (low quality of evidence/strong)	Anti-TNF if refractory to steroids, TP, or MTX (moderate quality of evidence/strong)	Combination therapy more effective than monotherapy (high quality of evidence/strong)	Not discussed	Not discussed	Not discussed	Anti-TNF or TP (moderate quality/conditional)	Antibiotics in low-risk disease (no grade)	Indirect evidence to support in highest risk patients (no grade)	Yes	Yes
Asia Pacific Association of Gastroenterology	Steroids (I, A)	Anti-TNFs and surgery (III, C)	Not discussed	Antibiotics (III, C)	Not explicitly discussed	Not explicitly discussed	Not discussed	Not discussed	Not discussed	No	No
National Institute for Health and Care Excellence	Steroids to induce remission (no grade); AZA/6-MP to add-on to steroids (no grade)	Anti-TNFs if has not responded to steroids, AZA, or 6-MP (no grade)	Not discussed	Antibiotics, drainage, immunosuppressants (no grade)	Infliximab after no response to conventional therapy (no grade)	Not discussed	AZA/6-MP in patients with adverse prognostic factors (no grade); consider 5-ASAs (no grade)	Not discussed	Not discussed	No	No
Canadian Association of Gastroenterology	Not explicitly discussed	Not explicitly discussed	Not explicitly discussed	Antibiotics (very low quality/conditional) + anti-TNFs (very low quality/strong)	Not discussed	When starting anti-TNF, recommend combining with TP/MTX (low or very low quality/conditional)	Not discussed	Not discussed	Not discussed	No	No
European Crohn’s and Colitis Organisation	Steroids to induce remission (high quality of evidence); TP/6-MP for maintenance (high/low quality of evidence)	Anti-TNF to induce remission if refractory to steroids (high quality of evidence); anti-TNF if severe disease for maintenance (very low quality of evidence)	If remission achieved with combination therapy, continue as maintenance (high quality of evidence)	Antibiotics for simple (low quality); infliximab or adalimumab for complex, plus antibiotics (high/moderate quality)	TP or anti-TNF for simple (low quality)	Can consider combination therapy with anti-TNF and TP in complex disease to enhance anti-TNF effect (very low quality)	TP (moderate quality) or anti-TNF (moderate quality)	Antibiotics (high quality); high dose mesalazine in isolated ileal resection (moderate quality)	Not discussed	Yes	Yes
Korean Association for the Study of Intestinal Diseases	Steroids to induce remission (moderate quality of evidence/strong); TP for maintenance in steroid-induced remission (moderate quality of evidence/strong)	Anti-TNF to induce remission if refractory to steroids (high quality of evidence/strong); anti-TNF for maintenance in anti-TNF-induced remission (high quality of evidence/strong)	Combination therapy more effective if anti-TNF used to induce remission (moderate quality of evidence/conditional); can continue combination or use anti-TNF monotherapy for maintenance (low quality of evidence/weak)	Antibiotics for simple (low quality/strong); infliximab or adalimumab for complex, plus antibiotics (high to moderate quality/strong)	Not discussed	Not discussed	5-ASA (high quality/conditional); TP in high risk of recurrence group (high quality/conditional); anti-TNF (moderate quality/conditional)	Not discussed	Not discussed	Yes	Yes

TNF, tumor necrosis factor; 5-ASA, 5-aminosalicylic acid; AZA, azathioprine; 6-MP, 6-mercaptopurine; TP, Thiopurine; MTX, methotrexate.

### Age of guidelines and expected updates

The mean age of guidelines amongst all societies is 5 years (standard deviation* *=* *2.6 years). Guideline age ranged from 1 year (written in 2019) to 11 years (written in 2009). Three societies included in the initial analysis included timelines for future updates or reviews of their guidelines (NICE, CAG, and Japanese Society of Gastroenterology).

## Discussion

The World Health Organization (WHO) has noted that guidelines are meant to advise physicians on treatments for their patients and to create a safer medical system [45]. In order to do this, it follows that guideline recommendations should be based on strong evidence quality and updated at regular intervals. In addition, author COI should be explicitly denoted. Finally, and most idealistically, these recommendations should also be consistent between different organizations. In 2013, it was found that nearly 50% of IBD guidelines were based on expert opinion and low-quality supporting evidence [4]. Unfortunately, little has changed, as our current analysis shows that almost 50% of UC and CD guidelines continue to be based on low- or very-low-quality evidence. With regard to COI, it is reassuring that nearly all international societies are now publicly documenting author COI—a major change compared to 2013 [4]. However, bias still continues to be a concern given that 50% of first authors and 64% of senior guideline authors had COI. When comparing UC and CD recommendations between societies, they continue to vary in both content and levels of supporting evidence. Finally, there is a significant dearth of societies documenting timelines for guideline revision or update—one of the hallmarks of the IOM CPG recommendations [2].

Poor evidence quality behind CPGs continues to be a challenge. Our 2013 study, as well as this current study, confirmed similar findings [4, 9–11]. Overall, societies have increased the use of a grading system to evaluate the evidence with increasing use of the preferred GRADE methodology. The underlying quality of the evidence is rooted in the available studies and data, though this is variably reported in CPGs. While this cannot be changed, the findings in this study of low-quality evidence should stimulate the need for better-designed primary studies to assist in guideline development. Additionally, differences in recommendations across societies based on the same evidence may reflect the difficulty in interpreting low-quality data and differences in healthcare systems and resources internationally. 

COI in both guideline development and clinical medicine continue to be a prominent issue, as they can lead to distrust of guidelines and prevent adequate and unbiased patient care. Highlighting the importance of COI in medicine, JAMA presented a theme issue in May 2017 on the topic [46]. Although it is common for clinical experts to have relationships with industry, transparency and consistency regarding the reporting of potential COI are critical. Compared to our 2013 study in which only ∼50% of CPGs commented on COI, we found that 86% of reviewed guidelines reported potential COI [4]. However, when reported, there were significant inconsistencies in the ways in which societies reported COI. In a study of 11 IBD CPGs, Grindal *et al.* [47] found that, while 62% of authors reported COI, there was significant variability depending on the country or region from which the guideline originated. In addition, only 23% of guidelines adhered to National Academy of Medicine standards for reporting COI [47]. The presence of ongoing COI highlights the importance for having a process to review the COI before the guideline is developed. In addition to having a system by which COI are reviewed before the guideline is developed, all COI should be reviewed by an external panel to aid in minimizing the influence of COI on CPG development. Ultimately, the purpose of COI transparency is to protect the integrity of professional judgment and improve standards of patient care.

As noted previously, one of the IOM standards is the creation of a schedule for guideline updates [2]. In our study, there were a limited number of societies that reported a plan and schedule for review of their guidelines. There is a lack of primary literature on this topic, though a 2014 study noted that a majority of guideline handbooks do not provide guidance on the CPG-updating process including literature search, evidence selection, and external review [48]. When guidelines do not maintain updated recommendations, they are ultimately rendered less relevant and prevent the advancement of patient care. For example, only 43% of reviewed guidelines remarked on the use of anti-integrins and anti-interleukins in CD management. When guidelines are not updated regularly, physicians are forced to seek alternate resources to clarify treatment options. Only actively updated guidelines will create better and safer patient care.

We note three limitations of this study. First, multiple systems were used to grade the quality of evidence by international societies. To allow uniform analysis of the level of evidence supporting the recommendations, we merged the grading systems into one system, based on the GRADE system of evidence quality. To limit potential bias, this was performed by two authors. Second, guidelines included in this analysis were required to be specifically regarding UC or CD. Thus, guidelines under the general heading “inflammatory bowel disease” were left out, which may be a substantial number. Nonetheless, we feel that these results would likely be applicable to those recommendations given that the same societies included in this study also publish these more “non-specific” guidelines. Furthermore, IBD guidelines published by other societies may exist, but were not considered in this analysis. Lastly, when assigning a low/very low level of evidence to recommendations without any supporting evidence, only one level was assigned even if multiple recommendations were included in one sentence. This potentially underestimates the total number of recommendations with ungraded evidence.

In summary, the majority of IBD CPG recommendations are based on low- and very-low-quality evidence, as per our GRADE-based system. This has unfortunately not changed significantly compared to 2013. Reassuringly, COI are reported much more frequently compared to 2013, though this is not a universal practice. Additionally, management recommendations vary between societies for both UC and CD. Few societies report a timeline for review of their guidelines and updates. This study continues to highlight the need for improving the development of IBD CPGs. Recommendations would be improved by stronger supporting evidence, agreement between societies, up-to-date recommendations, and transparency regarding all potential COI in the development process.

## Authors’ Contributions

Study concept and design: J.D.F. Acquisition of data: A.G., R.S., and G.H. Analysis and interpretation of data: A.G., R.S., G.H., and J.D.F. Drafting of the manuscript: A.G., R.S., G.H., and J.D.F. Critical revision of the manuscript for important intellectual content: A.G., R.S., G.H., and J.D.F. Statistical analysis: A.G. Study supervision: J.D.F. Each author has approved the final draft of this manuscript.

## Funding

No financial support was provided for this manuscript.

## Conflict of Interest

None declared.
